# Obstruction level associated with outcome in hypoglossal nerve stimulation

**DOI:** 10.1007/s11325-021-02396-y

**Published:** 2021-06-05

**Authors:** Markus Wirth, Maximilian Bautz, Franziska von Meyer, Benedikt Hofauer, Ulrich Strassen, Clemens Heiser

**Affiliations:** Department of Otolaryngology - Head and Neck Surgery, Technical University of Munich, Klinikum rechts der Isar, Hals-Nasen-Ohren-Klinik, Ismaninger Straße 22, 81675 Munich, Germany

**Keywords:** Manometry, OSA, Selective hypoglossal nerve stimulation, Obstruction level

## Abstract

**Purpose:**

Selective hypoglossal nerve stimulation (sHNS) constitutes an effective surgical alternative for patients with obstructive sleep apnea (OSA). sHNS results in tongue protrusion and consecutive alleviation of obstructions at the tongue base level (lower obstructions). Furthermore, obstructions at the soft palate level (upper obstructions) may be prevented through palatoglossal coupling as seen on sleep endoscopy. However, it has not been studied if the distribution of obstruction level during a whole night measurement is a relevant factor for the treatment outcome.

**Methods:**

Obstruction levels were measured with a manometry system during a whole night of sleep in 26 patients with OSA (*f* = 1, *m* = 25; age 59.4 ± 11.3; BMI = 29.6 ± 3.6) either before (*n* = 9) or after sHNS implantation (*n* = 12). Five patients received a measurement before and after implantation. Obstructions were categorized into velar (soft palate and above), infravelar (below soft palate), and multilevel obstructions. An association between obstruction level and treatment outcome was calculated.

**Results:**

The mean distribution of preoperative obstruction level could be divided into the following: 38% velar, 46% multilevel, and 16% infravelar obstructions. Patients with a good treatment response (defined as AHI < 15/h and AHI reduction of 50%) had fewer preoperative velar obstructions compared to non-responder *(*17% vs. 54%, *p*-value = 0.006)*.* In patients measured after sHNS implantation, a significantly higher rate of multilevel obstructions per hour was measured in non-responders (*p*-value = 0.012).

**Conclusions:**

Selective hypoglossal nerve stimulation was more effective in patients with fewer obstructions at the soft palate level. Manometry may be a complementary diagnostic procedure for the selection of patients for HNS.

## Introduction

About 20% of women and 50% of men suffer from moderate to severe sleep disordered breathing (SDB) according to a recent population-based study [1]. Most patients with SDB suffer from obstructive sleep apnea (OSA) [2]. Patients complain of excessive daytime sleepiness and cognitive deficits [3]. OSA also has a high societal relevance since approximately 20% of car accidents are related to sleep deprivation of which OSA is a main cause [4]. In addition, OSA is associated with secondary diseases especially of the cardiovascular system such as hypertension, coronary artery disease, and cardiac arrhythmias [5]. Several treatment options exist for patients with OSA ranging from conservative methods such as positive airway pressure (PAP) therapy and mandibular advancement devices to surgical interventions [6, 7]. For patients with poor compliance to conservative therapies and moderate to severe OSA, selective hypoglossal nerve stimulation (sHNS) may constitute an effective surgical alternative [8].

In sHNS, the main pharyngeal airway dilatory muscle and tongue protrudor is activated to prevent airway collapse during sleep [9]. Different stimulation techniques have been developed ranging from the activation of proximal sectors to distal fibers of the nerve [10]. One system frequently implanted stimulates the branches of the hypoglossal nerve which are required for tongue protrusion and is termed selective hypoglossal nerve stimulation (sHNS) [11]. The stimulation is synchronized with the breathing cycle in this system [11]. In addition to a significant AHI reduction, sleep architecture was also improved in patients with OSA by using this kind of neurostimulation [12]. Established criteria for selecting patients for sHNS are apnea–hypopnea index (AHI) between 15 and 65/h, BMI ≤ 35 kg/m^2^ and the absence of a complete concentric collapse at the palate level during drug-induced sleep endoscopy (DISE). DISE is the most established method to detect the level and pattern of pharyngeal obstructions in the preoperative assessment for surgical OSA treatment [13]. DISE, however, requires sedation and is operator-dependent [14]. Obstructions can alternatively be monitored during whole night of natural sleep with a multisensory manometry system [15]. Sleep architecture is not influenced by the system [16]. Obstructions measured with this system can be divided into velar (soft palate), infravelar (below soft palate), and multilevel obstructions. Manometry can provide valuable information compared to DISE, since REM sleep does not occur in propofol sedation [17] and infravelar obstructions increase in REM sleep [18].

The aim of this study was to determine if the treatment response of sHNS is associated with the obstruction level detected with manometry.

## Material and methods

### Patient selection

**Fig. 1:**
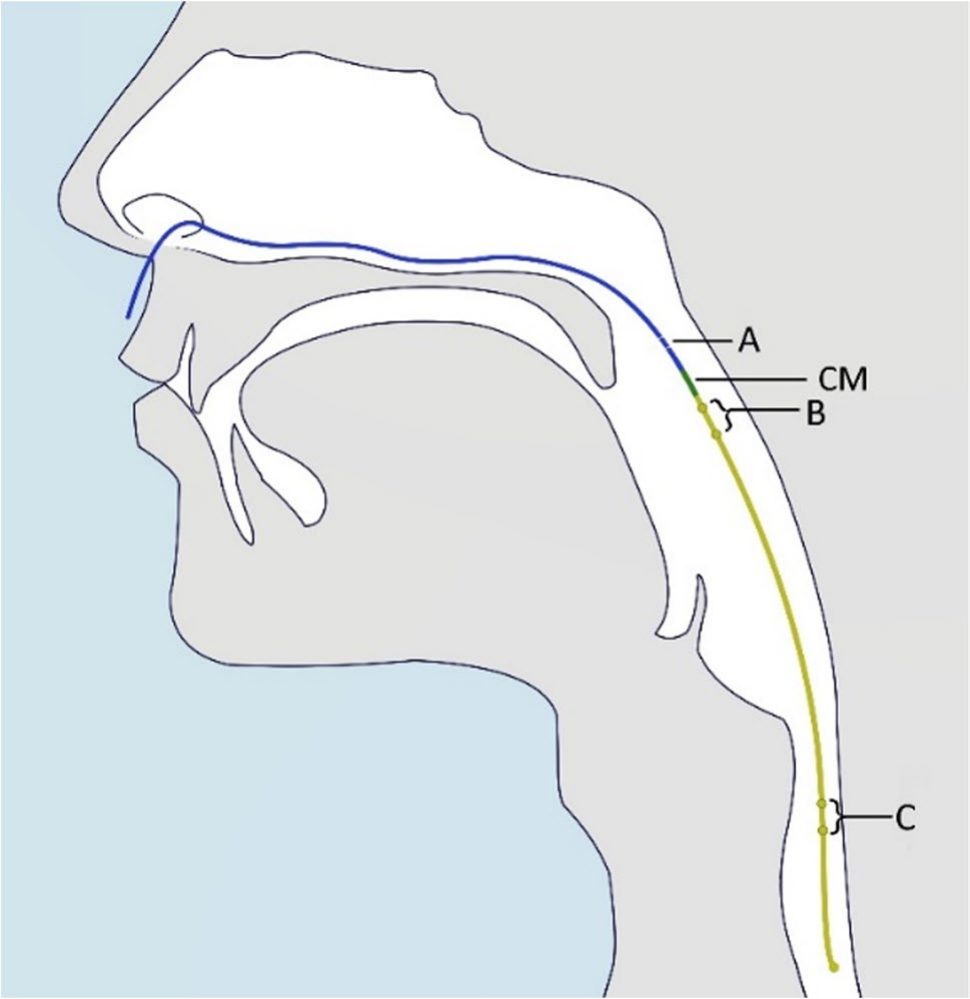
Manometry measurements with the ApneaGraph catheter. A Thermistor recording nasal airflow. B and C Sensors measuring local pressure level and airflow. CM, control mark for correct positioning

A total of 26 patients with OSA (1 woman and 25 men; mean age 59.4 years ± 11.3, ranging from 35 to 79 years) received manometry measurements (ApneaGraph Spiro or ApneaGraph 200 system, Spiro Medical, Bergen, Norway) in this study. In both systems, the measurement of the manometry was as described in Fig. 1; however, the size of the catheter was smaller in the newer ApneaGraph Spiro system (1.3 mm vs. 1.9 mm). The smaller catheter with the newer device resulted in less discomfort inserting the catheter and was therefore used as soon as available. For comparability, all manometry files were converted by Spiro Medical into the latest format. Patients presenting to the sleep laboratory for consultation (Department of Otolaryngology, Technical University of Munich) were enrolled if they were willing to participate and signed an informed consent. Fourteen patients were examined before implantation of selective hypoglossal nerve stimulation (sHNS), and of these patients, five patients were also measured after implantation with stimulation turned on. In addition, 12 patients were solely measured after implantation with stimulation turned on.

All patients received an in-lab polysomnography according to the AASM criteria [19] to confirm the diagnosis of OSA.

### Treatment response evaluation

The AHI reduction after 6 months of surgery or if not available (12 or 3 months) after implantation in comparison to the baseline-AHI (before surgery) was used to determine the treatment response. Successful treatment was defined as AHI < 15/h and a reduction of at least 50% (modified Sher criteria) to differentiate between treatment responder and non-responder [20].

### Selective hypoglossal nerve stimulation and tongue motion direction

All patients were selected for sHNS as described previously [12, 21] and implanted during the period from 2016 to 2018. Implantation was performed as recently published [11, 22]. The stimulation system was activated 1 month after implantation as described previously [23]. The second month after the implantation, stimulation was titrated using a polysomnography. Stimulation amplitude (V) and electrode configurations were individually titrated. Further control polysomnography or home sleep polygraphy were performed 3 to 12 months post-implantation followed by yearly control home sleep polygraphy.

After activation of the stimulation system, different tongue motions can be differentiated. The tongue motion with activated stimulation can be classified as bilateral (bilateral elongation and anterior displacement of the tongue), right protrusion (ipsilateral extension of the tongue with deviation to the left side), or mixed activation (includes every other kind of tongue motion such as shortening, retracting, or curling of the tongue) [24].

### Manometry examinations

The catheter manometry was performed to investigate the level of obstructions in the upper airway as described previously [18].

The AG deploys a thin catheter (AG Spiro 1,3 mm, AG 200: 1.9 mm) transnasally into the esophagus with the green control mark (CM) right below the base of the uvula for the correct positioning as depicted in Fig. 1. With this position secured, sensor A (thermistor) positioned in the posterior nasal cavity/anterior nasopharynx can correctly record nasal airflow. Sensors located in the oropharynx (B) and esophagus (C) measure the local pressure level, as well as airflow (B). A pulse oximeter attached to a finger of the patient measures oxygen saturation and heart rate. Recordings were analyzed manually for the whole recording period. The program used for this was Spiro Analysis Version 6.1.

To detect the level of obstructions, the ratio in amplitude between both pressure sensors was evaluated; Pressure ratio Rp [%] = ((B/(B + C)) × 100). If Rp was ≥ 60%, the event was scored as a velar (upper) obstruction (soft palate and above). If Rp was in the range of  40% ≤ Rp < 60 %, it was scored as a multilevel obstruction (both location with an equal amount of pressure). If Rp was in the range of < 40% , it was scored as an infravelar (lower) obstruction (between esophagus and soft palate). Every single apnea or hypopnea was classified according to the level in which it predominately occurred (at velum (velar), below the velum (infravelar) or at both levels (multilevel). The percentage of obstructions occurring in the different level was then calculated based on all obstructive events in each patient (apneas and hypopneas at one level/all apneas and hypopneas). Additionally for a sub-analysis, the percentage of apneas relative to all obstructive events (apneas at level/apneas + hypopneas at level) at the different level was calculated. For the comparison of the different cohorts (e.g., responder or non-responder), the mean percentage or median was used. After classification of the level of obstruction during the complete examination period, patients were scored as having predominantly velar, multilevel, or infravelar obstructions based on the obstruction level with the highest percentage of obstructions.

### Statistical analysis

All statistical tests were two-sided and significance was determined at a level of 5%. Statistical calculations were executed in SPSS version 25 (IBM, Ehningen, Germany). In normally distributed groups, comparison of distribution was performed with *T*-test, otherwise Wilcoxon signed-rank test, Mann–Whitney-*U* test, or Friedman tests were used. Linear regression was used to analyze the association between percentage of preoperative upper obstruction level and relative AHI reduction.

## Results

The characteristics of patients are depicted in Tables 1 and 2. Apnea–hypopnea index (AHI) of patients ranged from 18.0 to 70.6/h.

**Table 1:** Showing overall characteristics of study collective

Characteristic	Patients (*n* = 26)
Age (years)	59.4 ± 11.3
BMI (kg m^−2)^	29.6 ± 3.6
Females/males	1/25
AHI PSG preoperative (events per hour)	39.9 ± 16.0
Epworth Sleepiness Scale	10.1 ± 5.6

Values are mean ± SD

**Table 2:** Sleep variables in PSG prior to implantation

Sleep variable in % of TST				
Distribution of sleep stages	N1 13.0 [7.7, 28.8]	N2 68.0 [55.6, 80.1]	N3 5.2 [1.2, 10.1]	REM 7.0 [2.2, 14.0]
Distribution of sleep position	Supine 61.4 [22.9, 87.4]	Left 7.7 [0.0, 35.1]	Right 5.0 [0.0, 32.4]	Prone 0.1 [0.0, 2.1]

Values are median and IQR

### AHI reduction through implantation

**Fig. 2:**
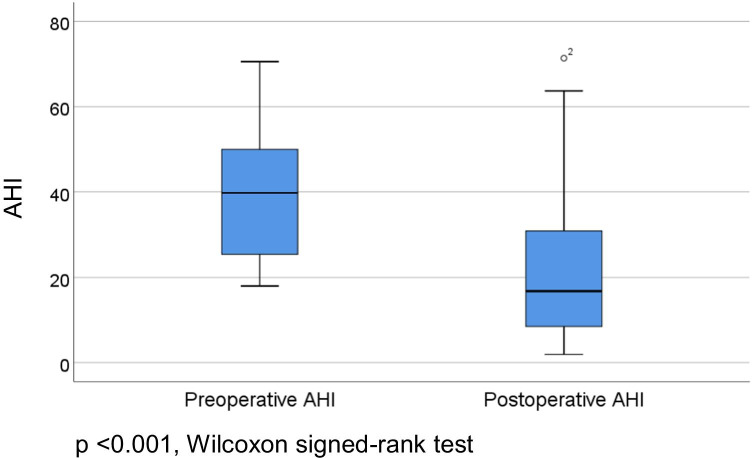
Depiction of preoperative and postoperative AHI in the patient cohort (3–12 months postoperative)

Median preoperative AHI was 39.8/h [25.4, 50.2] compared to 16.8/h [8.5, 31.4] in the postoperative measurement (3–12 months postoperative; *p* < 0.001; depicted in Fig. 2). Patients were divided into responder groups based on modified Sher criteria (AHI < 15 and a reduction of at least 50%). In the responder group (*n* = 11), AHI was significantly reduced after implantation (37.4/h [25.1, 40.9] vs. 8.3/h [4.0, 12.3], *p* = 0.003). In the non-responder group (*n* = 15), median preoperative AHI was 49.0/h [25.4, 62.3] compared to 30.0/h [20.2, 40.5] postoperatively (*p* = 0.020).

### Distribution of obstructions in the different level and association with AHI reduction

**Fig. 3:**
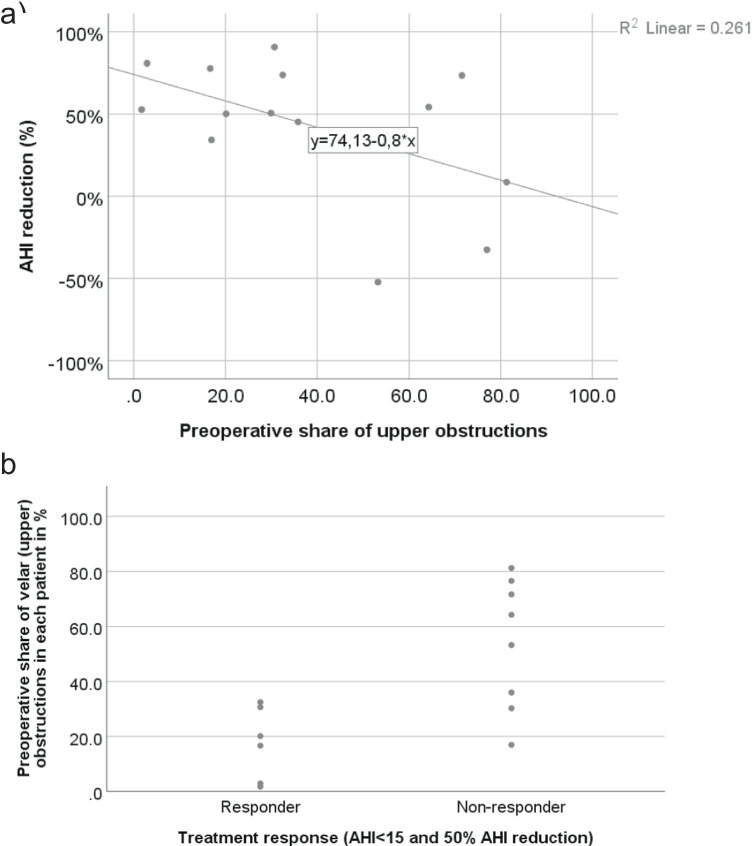
Depiction of association between proportion of preoperative velar (upper) obstructions and relative AHI reduction (**a**) and response status (**b**)

The mean distribution of preoperative obstruction level (*n* = 14) was 38% velar, 46% multilevel, and 16% infravelar obstructions. The mean preoperative AHI (*n* = 14) was lower in patients with a treatment response (defined as AHI < 15/h and AHI reduction of 50%) compared to non-responder (AHI 30.3 ± 4.1 vs. 45.8 ± 7.0, *p* = 0.106). Responders had a lower percentage of preoperative velar obstructions compared to non-responders (17% vs. 54%, *p*-value = 0.006, Table 3). In patients measured after sHNS implantation, the non-responders had a significantly higher rate of multilevel obstructions per hour (*p*-value 0.012, Table 4). A negative association between the proportion of preoperative velar obstructions and the relative AHI reduction was seen (*F* (1,12) = 4.24, *p* = 0.062) as depicted in Fig. 3.

**Table 3:** Distribution of preoperative obstruction level in responder vs. non-responder group (*n* = 14)

	Preoperative obstruction level in %		
Preoperative obstruction level	Responder (*n* = 6)	Non-responder (*n* = 8)	*p*-value
Velar	17.4 ± 13.2	53.8 ± 23.8	**0.006**
Multilevel	52.6 ± 6.1	40.4 ± 17.6	0.133
Infravelar	30.0 ± 15.5	5.8 ± 6.8	**0.002**

Values are mean ± SD. Significant values are shown in bold

**Table 4:** Distribution of events (apnea and hypopnea) per hour in the different obstruction level in the postoperatively measured cohort in responder vs. non-responder

Obstructive events (apnea and hypopnea) per hour in different obstruction levels in the postoperatively measured cohort			
Obstructive events per hour in obstruction level	Responder (*n* = 7)	Non-responder (*n* = 10)	*p*-value
Velar	8.0 [3.6, 11.2]	12.9 [2.9, 20.1]	0.417
Multilevel	5.7 ± 5.2	13.8 ± 6.2	**0.012**
Infravelar	0.6 [0.0, 2.5]	2.1 [0.6, 6.5]	0.193

All patients studied postoperatively (*n* = 17) were included (patients only measured postoperatively (*n* = 12) and the postoperative measurements of patients examined preoperatively and postoperatively (*n* = 5). Values are median and IQR or mean ± SD. Significant value is shown in bold

### Percentage of preoperative apneas relative to all obstructive events at respective level in responder vs. non-responder

In a further sub-analysis, only the percentage of preoperative apneas relative to all obstructive events at that level (apneas at level/apneas + hypopneas at that level) was analyzed in treatment responders and non-responders. Responders had a higher percentage of infravelar apneas relative to all obstructive events at infravelar location compared to non-responders (25.6 vs. 3.6%, *p* = 0.059, Table 5).

**Table 5:** Percentage of preoperative apneas relative to all obstructive events occurring at different obstruction location in responder vs. non-responder group (*n* = 14)

	Percentage of preoperative apneas relative to all obstructions at level in %		
Location of apneas	Responder (*n* = 6)	Non-responder (*n* = 8)	*p*-value
Velar	21.4 [0.0, 44.3]	17.6 [8.6, 40.0]	0.662
Multilevel	37.2 [25.0, 48.1]	28.7 [13.0, 49.1]	1.0
Infravelar	25.6 [15.8, 39.9]	3.6 [0.0, 18.4]	0.059

Values are median and IQR

### Association between postoperative tongue motion and percentage of residual velar obstructions

In the patient cohort with postoperative measurements of residual obstructions with sHNS turned on, four patients (24%) showed a right protrusion of the tongue and 13 patients (76%) bilateral tongue protrusion when stimulation turned on (measured 2 months postoperatively). No relevant difference in the percentage of residual velar obstructions was detected between patients with right vs. bilateral protrusion of tongue (52 ± 10% vs. 45 ± 29%, *p* = 0.50).

### Comparison of obstruction level pre- and postoperative

In 5 patients, preoperative and postoperative obstruction levels were also measured. In this group, 3 patients were non-responders. The rate of velar and infravelar obstructions per hour decreased only slightly between preoperative and postoperative (stimulation turned on) measurements (12.6 ± 11.8 vs 11.4 ± 10.6 per hour and 5.0 ± 4.8 vs. 4.3. ± 5.4 per hour). The rate of multilevel obstructions decreased from 13.6 ± 10.0 to 10.5 ± 6.8.

## Discussion

In this study, the obstruction level was analyzed via manometry throughout an entire night of natural sleep in patients with OSA either before (*n* = 14) or after treatment (*n* = 17) with sHNS to determine if treatment effectivenss depends on the anatomic location of obstructions.

Velar (upper level) obstructions were significantly higher in the preoperative measurements in non-responders (AHI > 50% and AHI > 15/h) compared to responders. Inversely, the percentage of infravelar (lower) obstructions was also significantly higher in responders. Furthermore, the preoperative percentage of apneas from all obstructive events at that level was analyzed with regard to treatment response. The relative proportion of apneas to overall obstructive events at the different level was not associated with treatment response. These results suggest that sHNS seems to be more effective on infavelar obstructions and that patients with a high percentage of velar obstructions may potentially be suboptimal cases for sHNS. In the evaluation of these results, it needs to be taken into consideration that a complete concentric collapse (CCC) was excluded prior to implantation with drug-induced sleep endoscopy (DISE) in all patients of our study cohort. A complete concentric collapse (CCC) is an important predictor for treatment success in sHNS [25] and occurs in 20–30% of patients with OSA [26]. Since a correlation between a complete concentric collapse and manometry findings has not yet been demonstrated, both DISE and manometry need to be performed before implantation.

To date, the level of obstruction has not been tested with manometry in patients with sHNS. Nonetheless, sHNS has been demonstrated in numerous studies to effectively reduce AHI [23]. This high degree of effectiveness requires a reduction of obstructions at all level. The resolution of velar obstructions in sHNS is believed to work via palatoglossal coupling and has been validated using drug-induced sleep endoscopy (DISE) [27]. Notwithstanding, Mulholland et al. validated with DISE that patients with worse baseline severity of collapse at the level of the lateral walls were accompanied by worse sHNS outcomes [28]. Contradictory findings remain concerning the relevance of the airway opening at the upper pharynx for sHNS effectiveness. The degree of opening of the retropalatal space with activated sHNS was associated with improved treatment outcome in one study [29]. In contrast, patients with significant airway improvement in the upper pharynx with mandibular advancement during DISE appeared less likely to succeed with sHNS [28]. These parameters are ideally assessed with sleep endoscopy, since the pattern and detailed anatomic location of obstruction are detected. However, DISE is an artificial examination for a limited time frame and not comparable to natural sleep. The alleviation of obstructions at the different level through sHNS measured during a whole night of natural sleep could therefore differ from DISE examination and may provide important additional information. Differing results between DISE and manometry have already been reported especially with regard to the detection of infravelar obstructions [30]. A potential reason for these observed differences could be the change in obstruction level occurring in REM sleep [18].

To further examine the effectiveness of sHNS on different levels of obstructions, sHNS patients were examined with manometry under stimulation. The rate of residual multilevel obstructions per hour with active stimulation was significantly higher in non-responders compared to that in responders. Also, relevant residual velar obstructions occurred in responders as well as non-responders. This could be in line with sHNS being less effective on velar and multilevel obstructions. Another explanation could be a higher sHNS efficiency on infravelar and multilevel obstructions in the responder group and an impaired efficiency at all obstruction levels in the non-responder group. In addition, the rates of obstructive events per hour in the different levels were tested preoperatively and postoperatively with active stimulation in five patients, and the highest reduction was seen in multilevel obstructions. However, the validity of this comparison is limited since there were three non-responders in this small group.

Because a bilateral protrusion of tongue base in DISE could be correlated with a better opening of the soft palate in a previous study [27], we analyzed the relationship between the percentage of velar obstructions and tongue protrusion (which leads to the protrusion of tongue base). In this study, 24% of patients in the postoperatively measured cohort showed a right tongue protrusion and 76% a bilateral tongue protrusion. The tongue motion was not associated with the percentage of residual velar obstructions with active stimulation. A possible explanation is that tongue motion observed in awake patients is not correlated with the bilateral protrusion of the tongue base visualized in DISE. Also, as outlined above, the relative share of velar obstructions in manometry during a whole night of sleep can differ from the opening of the soft palate in DISE. In addition, the baseline soft palate anatomy could be a confounding factor, especially since sHNS is a non-anatomically modifying surgery. Schwab et al. evaluated the soft palate volume on computed tomography on baseline and demonstrated that smaller soft palate volumes were associated with a favorable response [31].

In patients with insufficient treatment results with sHNS, several options exist to improve the therapy. For example, it has been suggested that palatal surgery could improve the outcome in non-responder with obstructions at velum level or oropharynx proven by DISE [32]. In reality, probably not all non-responder will profit from palatal surgery, and DISE seems not to discriminate the non-responder group well enough; as in the study by Steffen et al., almost 90% of suboptimal responders had a complete collapse at velum level [32]. Potentially, manometry could assist stratifying patients with suboptimal response profiting from palatal surgery and warrants further exploration. The preoperative localization of obstructions with manometry to select patients for uvulopalatopharyngoplasty (UPPP) was beneficial in one study [33]. However, a smaller study showed no association between the level of obstruction and patients’ UPPP outcome [34].

Despite thorough selection of patients, about 20–30% of patients are suboptimal responders according to the Sher criteria [23, 35]. It would be highly relevant to identify this patient group of suboptimal responders preoperatively. Several studies have been published on objective factors for the prediction of treatment success in HNS. Patients with lower therapeutic level of PAP were found to be more likely to have a treatment success in sHNS compared to patients with higher pressure requirements [36]. In the ADHERE registry, a lower BMI, higher age, and female gender were significant predictors of therapy response in a multivariate model [35, 37]. These are important findings but the difficulty remains how to stratify the “problematic” patient with high PAP pressure and BMI — potentially, manometry could be of use. Based on the findings in this study, it seems possible that the inadequate treatment response is at least partially caused by a high percentage of velar obstructions. Manometry could therefore possibly enrich the framework of objective factors for optimal HNS candidate selection. DISE would only be performed in preselected patients.

There are several limitations to our study. Most importantly the small sample size (due to the difficult recruitment of patients) restricts the detection of changes in subgroups and the results therefore need to be confirmed in a larger multi-center study. The percentage of non-responders in the study cohort is higher than reported in the literature [23, 38]. The high percentage in the postoperatively measured patients can be explained by more frequent visits of non-responders in our sleep laboratory, e.g., advanced titration. Also, all patients were preselected with DISE to rule out a complete concentric collapse. The overlap between patients with a complete concentric collapse at velum and a high percentage of velar obstructions can therefore not be discerned. Furthermore, the obstruction level could be subject to night to night variance since obstruction levels change with sleep stages. In subsequent projects, the night to night variance should be analyzed to verify our findings.

## Conclusion

In summary, this study emphasizes the soft palate area as critical for HNS success since a high percentage of velar obstruction was associated with treatment response. Manometry may be a complementary diagnostic procedure for the selection of patients for HNS.

## Abbreviations

AG: Apneagraph
AASM: American Academy of Sleep Medicine
AHI: Apnea–hypopnea index
BMI: Body mass index
CCC: Complete concentric collapse
CPAP: Continuous positive airway pressure
DISE: Drug-induced sleep endoscopy
ESS: Epworth sleepiness scale
EEG: Electroencephalography
EMG: Electromyography
EOG: Electrooculography
OSA: Obstructive sleep apnea
(s)HNS: (Selective) hypoglossal nerve stimulation
UPPP: Uvulopalatopharyngoplasty
